# Case Report: Colonic metastasis from mixed breast carcinoma masquerading as abemaciclib-associated diarrhea

**DOI:** 10.3389/fonc.2026.1802301

**Published:** 2026-07-15

**Authors:** Lin Lv, Xiaohui Wang, Xiaotao Zhu

**Affiliations:** Department of Breast and Thyroid Surgery, Affiliated Jinhua Hospital, Zhejiang University School of Medicine, Jinhua, Zhejiang, China

**Keywords:** abemaciclib, colonic metastasis, diagnostic overshadowing, mixed invasive ductal and lobular carcinoma, tumor heterogeneity

## Abstract

**Background:**

Invasive lobular carcinoma (ILC) demonstrates a distinctive propensity for gastrointestinal metastasis that differs from invasive ductal carcinoma (IDC). In mixed breast carcinomas, intratumoral heterogeneity poses challenges for disease surveillance and treatment assessment. Moreover, gastrointestinal adverse effects associated with CDK4/6 inhibitors, such as abemaciclib, may lead to diagnostic overshadowing, potentially delaying recognition of metastatic disease.

**Case description:**

A 68-year-old woman with mixed IDC and ILC underwent mastectomy following neoadjuvant chemotherapy. Histopathological evaluation revealed significant heterogeneity, with a luminal A IDC component and a triple-negative ILC component, the latter involving axillary lymph node metastases. During treatment with letrozole and abemaciclib, the patient developed progressive abdominal pain accompanied by rising carcinoembryonic antigen (CEA) levels, which were initially attributed to treatment-related toxicity. While bone metastases with a luminal phenotype remained radiographically stable, colonoscopy revealed an obstructing lesion in the descending colon. Biopsy confirmed metastatic carcinoma with a triple-negative profile (GATA3-positive, CDX2-negative), consistent with the primary ILC component and distinct from the bone metastases.

**Conclusions:**

This case highlights that persistent or evolving gastrointestinal symptoms during CDK4/6 inhibitor therapy warrant thorough evaluation for metastatic disease, particularly in patients with ILC. It also suggests that aggressive minor components in mixed tumors may have important clinical and prognostic implications. It emphasizes the importance of re-biopsy to identify phenotypic discordance and current therapeutic targets, informing timely and appropriate modifications to the treatment strategy.

## 1 Introduction

Breast cancer remains the most common malignancy among women globally. Invasive ductal carcinoma (IDC) constitutes the predominant histological subtype, whereas invasive lobular carcinoma (ILC) accounts for approximately 10–15% of cases (1). Although distant metastases most frequently involve the bone, lung, liver, and brain, metastatic behavior in mixed breast carcinomas is often complex and heterogeneous. The ILC component demonstrates a distinctive propensity for spread to the gastrointestinal tract, peritoneum, retroperitoneum, and female genital tract (2, 3). Colonic involvement is particularly uncommon, with reported rates of less than 2% among all patients with breast cancer (4). Notably, a recent review reported that only 8 of 169 (4.7%) cases of ILC metastasis to the gastrointestinal tract involved mixed IDC-ILC tumors, further underscoring the rarity of this presentation (5).

The introduction of cyclin-dependent kinase (CDK) 4/6 inhibitors, including abemaciclib, in combination with endocrine therapy has become the standard of care for hormone receptor–positive, human epidermal growth factor receptor 2–negative (HR+HER2−) advanced breast cancer, leading to substantial improvements in survival. This therapeutic approach is also increasingly applied in the adjuvant setting for patients with high-risk early-stage disease (6). However, the high incidence of diarrhea associated with abemaciclib, affecting more than 80% of treated patients, presents a significant challenge for clinical surveillance (7). In this context, gastrointestinal symptoms are frequently attributed to treatment-related toxicity, a phenomenon referred to as “diagnostic overshadowing,” which may delay the recognition of gastrointestinal metastatic disease.

This report describes a rare case of mixed breast carcinoma with pronounced intratumoral heterogeneity. The patient, diagnosed with concurrent IDC and ILC, developed luminal-type bone metastases together with an occult colonic metastasis showing a triple-negative phenotype during abemaciclib therapy. This case illustrates how abemaciclib-associated diarrhea may obscure symptoms of metastatic disease. It also highlights the value of re-biopsy in mixed histology tumors to enable timely and appropriate adjustments in therapeutic strategy.

## 2 Case description

A 68-year-old postmenopausal woman with no personal or family history of malignancy or inflammatory bowel disease presented to Jinhua Municipal Central Hospital. On March 24, 2024, a core needle biopsy established the diagnosis of IDC of the left breast with axillary lymph node involvement. The disease was clinically staged as cT1N1M0 (Stage IIA), luminal A subtype. Importantly, the serum carcinoembryonic antigen (CEA) level was significantly elevated at 100.3 ng/mL. ^18^F-fluorodeoxyglucose positron emission tomography–computed tomography (^18^F-FDG PET-CT) revealed multiple sclerotic skeletal lesions with increased FDG uptake throughout the body, raising suspicion of bone metastases. At the same time, no abnormalities were detected in other organs. The patient declined a confirmatory bone biopsy at that time.

Following multidisciplinary discussion, neoadjuvant chemotherapy was initiated, as distant metastasis had not been pathologically confirmed. Beginning April 2, 2024, she received the AC-T regimen (doxorubicin and cyclophosphamide followed by paclitaxel, administered every three weeks for eight cycles). Given the suspected bone involvement, denosumab was initiated every 4 weeks for skeletal protection. A repeat PET-CT after completion of chemotherapy on August 27, 2024, demonstrated stable skeletal lesions without evidence of new visceral disease. On September 20, 2024, the patient underwent a modified radical mastectomy of the left breast. Postoperative pathology revealed IDC with multifocal pleomorphic ILC. Response to neoadjuvant therapy was estimated as grade 2 according to the Miller–Payne criteria (8). All 21 excised axillary lymph nodes harbored metastatic carcinoma with treatment effect, histologically consistent with ILC. The pathological stage was ypT1N3M0 (Stage IIIC). Immunohistochemical analysis confirmed significant intratumoral heterogeneity: the IDC component was grade 2, luminal A subtype, whereas both the ILC component within the primary tumor and the nodal metastases were grade 3 and triple-negative. Adjuvant radiotherapy and endocrine therapy with letrozole were subsequently administered.

The patient’s CEA level declined to approximately 70 ng/mL after two chemotherapy cycles and remained at this plateau. Given the lack of further decline following surgery, persistent disease was suspected. A bone biopsy performed on October 28, 2024, confirmed metastatic carcinoma in the bilateral ilium and clavicles consistent with breast origin, demonstrating a luminal B phenotype. Germline BRCA1/2 testing revealed no pathogenic variants. On December 3, 2024, abemaciclib was added to the treatment regimen at a reduced dose of 100 mg twice daily because of the patient’s advanced age. During the initial two months of therapy, she developed grade 3 diarrhea according to the Common Terminology Criteria for Adverse Events (CTCAE), which improved to grade 1 with loperamide and dietary modification. Thereafter, bowel habits stabilized at grade 0–1, without blood or mucus, although intermittent, poorly localized, paroxysmal abdominal pain persisted in the absence of distension or vomiting. Other adverse events included grade 1 leukopenia, thrombocytopenia, and anemia. Due to limited tolerance, the abemaciclib dose was not escalated to 150 mg twice daily. Serial evaluations conducted every three months demonstrated no radiographic evidence of disease progression.

On June 6, 2025, the patient reported worsening episodic abdominal pain localized predominantly to the left lower quadrant, which was alleviated by defecation. Physical examination revealed a soft, non-distended abdomen without palpable masses, with only mild tenderness in the left lower quadrant and no rebound tenderness. There were no overt clinical signs of bowel obstruction. These symptoms were initially attributed to abemaciclib-related gastrointestinal toxicity.

By September 9, 2025, follow-up assessment showed a substantial increase in CEA to 141.8 ng/mL, raising concern for disease progression. However, breast and abdominal ultrasonography, chest and abdominal computed tomography, and bone scintigraphy failed to identify new metastatic lesions. Upper and lower gastrointestinal endoscopy were recommended; however, the patient opted for close surveillance, citing the invasive nature of the procedures, increased risks of infection and bleeding associated with leukopenia and thrombocytopenia, and reassurance provided by two previous PET-CT scans showing no gastrointestinal involvement.

**Figure 1:**
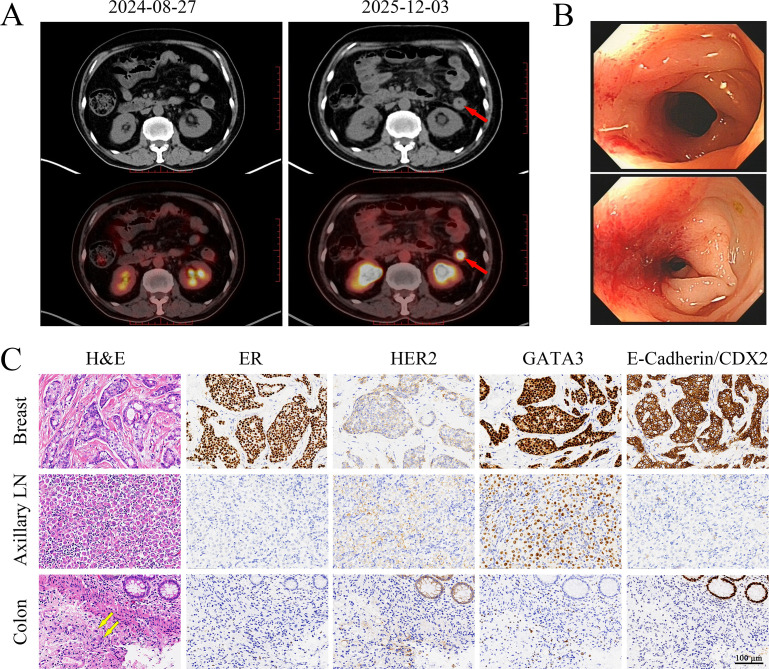
Pathological and imaging characteristics showing tumor heterogeneity and metastatic progression. **(A)** Serial PET-CT imaging. Comparison between August 2024 (left) and December 2025 (right). The follow-up scan reveals *de novo* circumferential wall thickening in the descending colon with increased FDG metabolism (SUVmax 8.9, red arrow). **(B)** Colonoscopic findings. Endoscopic view of the descending colon showing rigid bowel walls with luminal stenosis, without a clear mucosal mass. **(C)** Immunohistochemical comparison among tumor components. Top row (Breast, Primary IDC): H&E staining shows typical ductal structures. Tumor cells are ER-positive, HER2-negative, GATA3-positive, with retained E-cadherin membrane expression. Middle row (Axillary LN, Metastatic ILC): H&E staining shows pleomorphic tumor cells. Tumor cells are ER-negative, HER2-negative, and GATA3-positive, with loss of E-cadherin expression. Bottom row (Colon, Metastasis): H&E staining shows infiltration of atypical cells in the lamina propria (yellow arrows). Tumor cells are ER-negative, HER2-low, GATA3-positive, and CDX2-negative. PET-CT, positron emission tomography–computed tomography; FDG, fluorodeoxyglucose; SUVmax, maximum standardized uptake value; IDC, invasive ductal carcinoma; ILC, invasive lobular carcinoma; LN, lymph node; H&E, hematoxylin and eosin; ER, estrogen receptor; HER2, human epidermal growth factor receptor 2.

**Figure 2:**
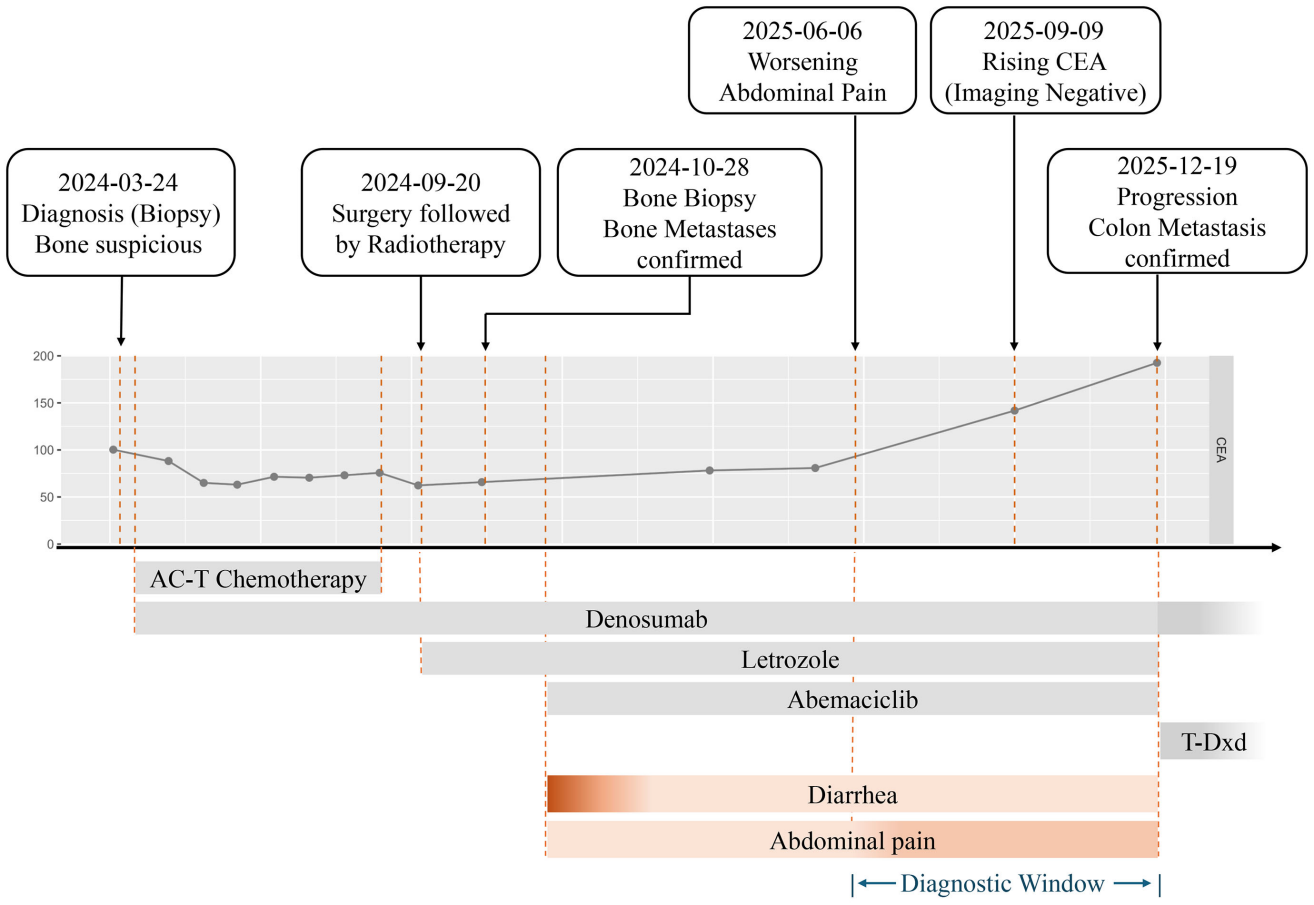
Longitudinal clinical timeline, CEA trend, and symptom evolution. The central line graph depicts changes in serum CEA levels (ng/mL) throughout the clinical course. Upper panel: Key diagnostic and therapeutic milestones. Lower panel: Duration of systemic treatments and corresponding gastrointestinal symptomatology. Diagnostic Window: Represents the critical period where the change in abdominal pain pattern and rising CEA were initially obscured by the known overlapping toxicity of abemaciclib and negative conventional imaging, potentially contributing to delayed detection of the colonic metastasis. CEA, carcinoembryonic antigen; T-DXd, trastuzumab deruxtecan.

On December 3, 2025, the CEA level rose further to 192.6 ng/mL. The patient continued to experience abdominal pain, diarrhea, and fatigue, with physical findings unchanged from previous visits. A repeat PET-CT demonstrated new focal wall thickening of the descending colon with increased FDG metabolism (maximum standardized uptake value [SUVmax]: 8.9; **Figure 1A**), suggestive of malignancy, while metabolic activity in the bone lesions had decreased compared with earlier scans. Colonoscopy performed on December 19, 2025, revealed a rigid, stenotic segment of the descending colon (**Figure 1B**). Histological examination of biopsy specimens showed sparse atypical cells infiltrating the lamina propria (**Figure 1C**). Extended immunohistochemical profiling demonstrated GATA3 positivity, CDX2 negativity, estrogen receptor negativity, and HER2 expression scored as 1+, supporting metastatic breast carcinoma consistent with the ILC component rather than primary colorectal adenocarcinoma. In response, abemaciclib and letrozole were discontinued. Given the HER2-low status, treatment was transitioned to trastuzumab deruxtecan (T-DXd). A detailed clinical timeline is summarized in **Figure 2**, highlighting the temporal relationship among CEA dynamics, therapeutic interventions, symptom evolution, and the eventual diagnosis.

## 3 Discussion

This case highlights three important clinical issues in the management of mixed breast carcinoma: diagnostic masking during CDK4/6 inhibitor therapy, the potential significance of aggressive minor components in mixed tumors, and the role of re-biopsy in guiding subsequent treatment decisions.

First, this case illustrates the risk of diagnostic masking when gastrointestinal symptoms arise during CDK4/6 inhibitor therapy. Diarrhea is a well-recognized adverse effect of CDK4/6 inhibitors, particularly abemaciclib, with reported all-grade incidence exceeding 80% and grade 3 events occurring in approximately 10% of patients. Abemaciclib-related diarrhea typically follows a predictable temporal pattern, emerging early during treatment, peaking within the first treatment cycle, and subsequently improving or becoming manageable with supportive care as therapy continues (9). In this case, although the patient initially achieved symptom control, a qualitative change in gastrointestinal complaints became evident after six treatment cycles, with nonspecific abdominal discomfort progressing to localized pain in the left lower quadrant. Such a shift in symptom pattern should be regarded as a clinically meaningful warning sign. Because gastrointestinal metastases from ILC often present as diffuse infiltrative disease resembling linitis plastica rather than discrete intraluminal masses, they may be missed on routine non-contrast computed tomography (10). Accordingly, in patients receiving prolonged CDK4/6 inhibitor therapy, persistent gastrointestinal symptoms or a change in symptom characteristics, particularly when accompanied by unexplained elevations in tumor markers such as CEA, should prompt consideration of underlying organic pathology rather than attribution to drug toxicity alone. This diagnostic consideration may also be relevant in other treatment settings associated with gastrointestinal toxicity, including tyrosine kinase inhibitors (11).

Second, this case highlights the potential clinical significance of aggressive minor components in mixed breast carcinoma, in the context of marked intratumoral heterogeneity (12). The primary tumor comprised both luminal A IDC and triple-negative ILC components. During endocrine therapy combined with CDK4/6 inhibition, the bone metastases with a luminal phenotype remained radiographically stable and showed reduced metabolic activity, whereas the later colonic lesion displayed a triple-negative phenotype consistent with the ILC component. Although a definitive evolutionary relationship cannot be established from a single case, this pattern suggests that a quantitatively smaller but biologically aggressive component may have disproportionate clinical relevance. In retrospect, the preoperative core needle biopsy did not capture the triple-negative component, underscoring the limitations of conventional sampling in heterogeneous tumors; larger-volume sampling may improve detection of phenotypically distinct components in selected cases (13). This sampling limitation may have therapeutic relevance, as earlier recognition of the triple-negative component might have influenced treatment considerations, although any retrospective inference regarding alternative management remains speculative. Even when multiple components are recognized, clinical decision-making often prioritizes the luminal subtype due to its therapeutic tractability and numerical dominance. However, this case suggests that such an approach may underestimate the prognostic impact of aggressive minor components. Careful attention to these minor components may be warranted, as they may have important implications for disease behavior and therapeutic planning (14).

Finally, this case underscores the value of re-biopsy in guiding subsequent treatment decisions. Endoscopic biopsy with extended immunohistochemical profiling (GATA3-positive, CDX2-negative) confirmed the colonic lesion as metastatic breast carcinoma, excluding primary colorectal cancer. Although the precise origin of this lesion cannot be determined with certainty, receptor discordance between primary and metastatic breast cancer is well documented and has direct clinical relevance. Reported rates of receptor conversion range from 10% to 50% in recurrent or metastatic disease (15). Although the metastatic lesion was estrogen receptor–negative, it showed low HER2 expression. Based on the pivotal DESTINY-Breast04 trial, T-DXd has demonstrated significant efficacy in patients with HER2-low metastatic breast cancer (16). Reliance on the historical luminal phenotype of the primary tumor or bone metastases would therefore have resulted in suboptimal treatment. Re-biopsy of the newly emerged metastatic site enabled reassessment of tumor phenotype and supported a clinically relevant change in therapy.

This study is limited by being a single case report, which precludes statistical generalization. Genomic profiling was not performed, and therefore clonal relationships among the primary tumor and metastatic lesions could not be definitively established. Bone metastases were not pathologically confirmed before surgery because the patient initially declined biopsy. If bone metastases had been confirmed at that time, the disease would have been managed as *de novo* stage IV HR+HER2− breast cancer based on the available receptor profile, with endocrine therapy plus a CDK4/6 inhibitor generally considered first-line systemic treatment and surgery reconsidered in a multidisciplinary context. Although earlier CDK4/6 inhibitor-based therapy might have influenced the subsequent clinical course, this uncertainty does not alter the central observation that gastrointestinal toxicity during abemaciclib treatment may mask colonic metastasis. The strength of this report lies in its comprehensive pathological, immunohistochemical, biomarker, and imaging documentation of a clinically informative case involving diagnostic masking, phenotypic heterogeneity, and treatment-relevant re-biopsy findings.

## 4 Conclusion

This case emphasizes the importance of improved clinical vigilance in the management of mixed breast carcinoma. To our knowledge, this appears to be a rare report of colonic metastasis whose recognition may have been delayed by abemaciclib-associated gastrointestinal symptoms. Clinicians should remain alert to the risk of diagnostic anchoring and avoid routinely attributing gastrointestinal symptoms to CDK4/6 inhibitor–related toxicity, particularly when symptoms persist, evolve in character, or are accompanied by unexplained elevations in CEA levels. Moreover, this case illustrates how intratumoral heterogeneity may contribute to divergent metastatic behavior and heterogeneous therapeutic responses within a single patient. Accordingly, whenever clinically feasible, re-biopsy of metastatic lesions should be strongly considered as a critical component of precision oncology, enabling timely and appropriately tailored treatment modifications.

## 5 Patient perspective

After taking abemaciclib for some time, I was initially relieved when my diarrhea finally subsided and became manageable. However, the persistent abdominal pain was confusing, as it was assumed to be another lingering medication-related side effect. After doctors suggested a colonoscopy, I was hesitant at first due to its invasive nature, but I am glad I agreed. Finding the true cause of my pain and knowing there is a new targeted treatment option available has given me renewed hope.

## Data Availability

The original contributions presented in the study are included in the article/supplementary material. Further inquiries can be directed to the corresponding author.
